# Mitochondrial OMA1 and OPA1 as Gatekeepers of Organellar Structure/Function and Cellular Stress Response

**DOI:** 10.3389/fcell.2021.626117

**Published:** 2021-03-25

**Authors:** Robert Gilkerson, Patrick De La Torre, Shaynah St. Vallier

**Affiliations:** ^1^Department of Biology, The University of Texas Rio Grande Valley, Edinburg, TX, United States; ^2^Clinical Laboratory Sciences/Department of Health and Biomedical Sciences, The University of Texas Rio Grande Valley, Edinburg, TX, United States

**Keywords:** mitochondria, apoptosis, OPA1, OMA1, development

## Abstract

Mammalian mitochondria are emerging as a critical stress-responsive contributor to cellular life/death and developmental outcomes. Maintained as an organellar network distributed throughout the cell, mitochondria respond to cellular stimuli and stresses through highly sensitive structural dynamics, particularly in energetically demanding cell settings such as cardiac and muscle tissues. Fusion allows individual mitochondria to form an interconnected reticular network, while fission divides the network into a collection of vesicular organelles. Crucially, optic atrophy-1 (OPA1) directly links mitochondrial structure and bioenergetic function: when the transmembrane potential across the inner membrane (ΔΨ_m_) is intact, long L-OPA1 isoforms carry out fusion of the mitochondrial inner membrane. When ΔΨ_m_ is lost, L-OPA1 is cleaved to short, fusion-inactive S-OPA1 isoforms by the stress-sensitive OMA1 metalloprotease, causing the mitochondrial network to collapse to a fragmented population of organelles. This proteolytic mechanism provides sensitive regulation of organellar structure/function but also engages directly with apoptotic factors as a major mechanism of mitochondrial participation in cellular stress response. Furthermore, emerging evidence suggests that this proteolytic mechanism may have critical importance for cell developmental programs, particularly in cardiac, neuronal, and stem cell settings. OMA1’s role as a key mitochondrial stress-sensitive protease motivates exciting new questions regarding its mechanistic regulation and interactions, as well as its broader importance through involvement in apoptotic, stress response, and developmental pathways.

## Introduction

The mitochondria of mammalian cells are increasingly understood to be a highly dynamic organellar network, using opposing fission and fusion pathways to homeostatically balance mitochondrial organization and bioenergetic function. Fusion of the inner membrane, mediated by optic atrophy-1 (OPA1), is a stress-sensitive mechanism of mitochondrial dynamic homeostasis, controlled by the OMA1 metalloprotease. Loss of OPA1 fusion causes the collapse of the mitochondrial network and promotes apoptosis. Our current understanding of OMA1’s crucial role in mitochondrial dynamics demonstrates that this proteolytic mechanism has broad importance to cell stress response, raising exciting new questions regarding OMA1’s mechanistic regulation, participation in apoptosis, and novel roles in differentiation and development.

## Mitochondrial Dynamics and Bioenergetic Function

**FIGURE 1:**
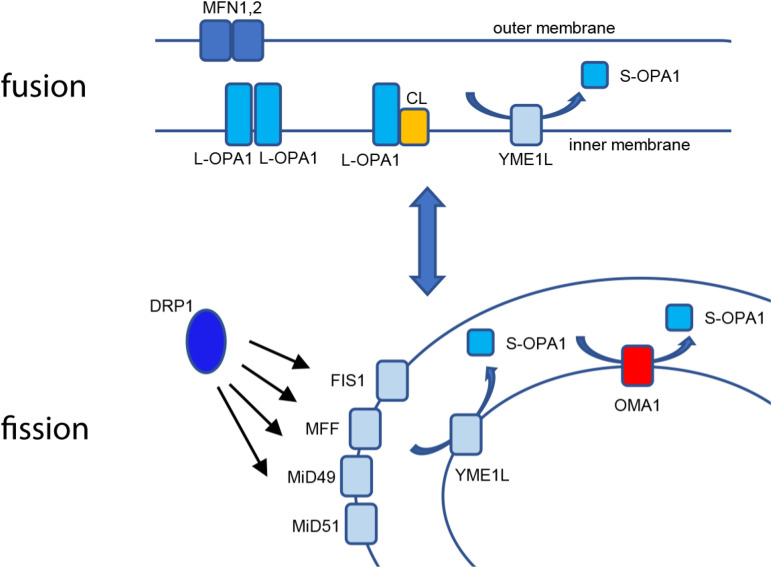
Mitochondrial fusion and fission. Fusion of the mitochondrial outer membrane is carried out at MFN1 and 2, while L-OPA1 maintains continuity of the inner membrane, either by homotypic interaction or by binding cardiolipin (CL). YME1L constitutively cleaves L-OPA1, resulting in basal S-OPA1. Fission is mediated by recruitment of cytosolic DRP1 to the outer membrane using actin-dependent dynamics, where it is bound by mitochondrial binding partners FIS1, MFF, MiD49, and MiD51. When activated, OMA1 cleaves L-OPA1 to S-OPA1 in cooperation with YME1L for accumulation of fusion-inactive S-OPA1.

From their earliest descriptions as “thread-like granules” giving rise to their designation as mitochondria, these organelles have undergone a profound reappraisal to our current understanding of mitochondrial structure/function as a highly responsive, dynamic network. Early work using light microscopy allowed investigators to appreciate the filamentous nature of the mitochondrial network (Ernster and Schatz, 1981). The advent of thin-section transmission electron microscopy, including seminal works by Palade (1952) and Sjostrand (1953), advanced the understanding of the multimembrane organization of the organelle, in which the outer membrane envelopes the organelle, while the inner membrane opposes the outer membrane at the periphery of the organelle but also is organized into tubules or folds (cristae) that extend through the interior matrix compartment of the organelle (Frey and Mannella, 2000). This internal organization provides a maximized surface/area ratio as the site of oxidative phosphorylation (OXPHOS). The multisubunit electron transport Complexes I–IV utilize NADH and FADH_2_ to establish a proton-motive transmembrane potential (ΔΨ_m_). This electrochemical gradient then powers the F_1_F_0_ ATP synthase, which uses ΔΨ_m_ to drive synthesis of ATP from ADP and Pi (DiMauro and Schon, 2003). As such, the mitochondrial inner membrane is highly specialized for bioenergetics, with structural adaptations to maximize metabolic function. Thin-section TEM images, while highly informative, also gave rise to the somewhat erroneous canonical textbook view of mitochondria as static, bean-shaped “batteries,” with one or two of these organelles tucked away at the back of the cell. Advances in fluorescence and imaging technology led to a reappraisal of mitochondrial ultrastructure, revealing the pleiomorphic, dynamic nature of the mitochondrial structure as a highly interconnected reticular network, a population of isolated vesicular organelles, or a balance of the two states (Amchenkova et al., 1988; Rizzuto et al., 1998). At the same time, the specific factors governing these elegant organellar dynamics emerged, demonstrating a set of sensitive, responsive factors that govern mitochondrial structural dynamics by balancing both organellar fission and fusion events. Mitochondria undergo fission by the recruitment of the cytosolic dynamin-related protein-1 (DRP1) to the mitochondrial outer membrane, which forms a multimeric collar around the mitochondrial tubule and constricts for membrane scission (Smirnova et al., 2001). DRP1 is bound at the outer membrane by an array of interacting partners, including mitochondrial fission protein-1 (Fis1), mitochondrial fission factor (Mff) (Gandre-Babbe and van der Bliek, 2008), and mitochondrial dynamic factors of 49 kDa (MiD49) and 51 kDa (MiD51) (Loson et al., 2013; Palmer et al., 2013). A variety of cellular stimuli, including ΔΨ_m_ uncouplers, low serum, or pro-apoptotic stimuli such as staurosporine and etoposide (Loson et al., 2013), cause phosphorylation-sensitive activation of DRP1’s mitochondrial recruitment, leading to fission of the mitochondrial network (Dickey and Strack, 2011; Ji et al., 2015). Figure 1 intriguing mechanistic questions remain for the mitochondrial fission machinery; for example, Voeltz and coworkers found that dynamin-2 (Dyn2) plays a role in completing separation of the two organelles during fission (Lee et al., 2016), while Raimundo’s group found that DRP1 was sufficient to complete fission without Dyn2 and additional dynamin partners (Fonseca et al., 2019). Fission is balanced by a separate set of factors controlling mitochondrial fusion. Mitofusins 1 and 2 maintain fusion of the mitochondrial outer membrane, independent of bioenergetic function (Santel and Fuller, 2001; Chen et al., 2003). Fusion of the mitochondrial inner membrane, conversely, requires an intact ΔΨ_m_ and is mediated by OPA1.

## OPA1 and OMA1: Stress-Sensitive Mitochondrial Fusion

**FIGURE 2:**
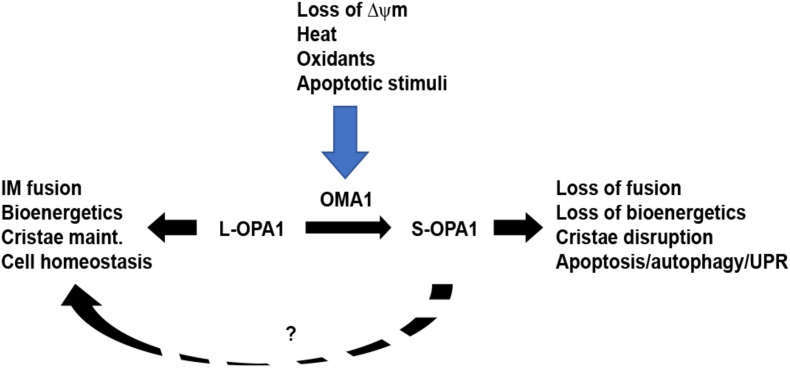
OMA1 controls stress-sensitive cleavage of long OPA1 isoforms. Under steady-state conditions, mitochondria maintain a balance of long, fusion-active L-OPA1 and short, fusion-inactive S-OPA1 isoforms. While YME1L (not shown) causes constitutive cleavage of L-OPA1 to produce steady-state S-OPA1, the OMA1 metalloprotease is activated by a range of stress stimuli. Upon activation, OMA1 cleaves L-OPA1, causing accumulation of S-OPA1, mitochondrial fragmentation, and loss of mitochondrial bioenergetics. This, in turn, primes the cell for increased stress response via mechanisms including apoptosis, autophagy, and unfolded protein response. While S-OPA1 isoforms cannot mediate mitochondrial inner membrane fusion, they may contribute to maintaining mitochondrial homeostasis (dashed line, ?).

Optic atrophy-1 is an essential GTPase responsible for fusion of the mitochondrial inner membrane. There are a total of eight mRNA splice variants of *OPA1*, processed for tissue-dependent expression (Ishihara et al., 2006). Following translation, mitochondrial importation, and insertion into the inner membrane, the high concentration of cardiolipin (CL) allows for CL-OPA1 tethering or OPA1:OPA1 homotypic association, followed by GTP-dependent membrane fusion (Ban et al., 2017; Figure 1). Recent crystallographic and cryo-EM analyses of OPA1’s yeast homolog Mgm1, provide new insights into how OPA1 remodels the inner membrane to mediate fusion (Faelber et al., 2019; Yan et al., 2020). In addition to facilitating inner-membrane fusion, OPA1 promotes dimerization of ATP synthase (Patten et al., 2014) and interacts with the multisubunit Mitochondrial contact site and Cristae Organizing System (MICOS) to help mediate cristae organization in addition to remodeling of the inner membrane (Hu et al., 2020; Stephan et al., 2020). Western blot analysis shows five distinct protein isoforms of OPA1: two long (L-OPA1) isoforms that mediate inner-membrane fusion and three short (S-OPA1) fusion-inactive isoforms. This pattern results from cleavage at OPA1’s S1 and S2 sites, which release S-OPA1 into the intermembrane space (Griparic et al., 2007; Guillery et al., 2008). Basal levels of S-OPA1 are produced by constitutive cleavage of OPA1 at the S2 site (Griparic et al., 2004), producing a steady-state balance of long and short OPA1 isoforms. Intriguingly, treatment of cells with some pharmacological inhibitors of mitochondrial OXPHOS, such as valinomycin, oligomycin, or carbonyl cyanide *m*-chlorophenylhydrazine (CCCP), but not others (rotenone, cycloheximide, antimycin A), causes inducible cleavage of L-OPA1 (Griparic et al., 2007; Guillery et al., 2008), demonstrating that L-OPA1 is specifically processed in response to loss of ΔΨ_m_. This loss of fusion causes unopposed mitochondrial fission and fragmentation of the mitochondrial network (Figure 2). Moreover, the two distinct pathways may impact each other mechanistically: fission-active Fis1 binds to MFN1 and 2, as well as OPA1 (Yu et al., 2019). These findings suggest that the interactions of the two distinct organelle remodeling pathways, both with each other and with bioenergetic function, are more complex than previously appreciated. Consistent with this, fission and fusion events both occur at sites of mitochondria-endoplasmic reticulum (ER) contact (Friedman et al., 2011; Abrisch et al., 2020), indicating a higher-order spatial coordination of fission and fusion pathways.

The overlapping with m-AAA protease (OMA1) metalloprotease was concurrently identified as the stress-sensitive protease responsible for L-OPA1 cleavage upon dissipation of ΔΨ_m_: Langer’s group, having previously identified OMA1 in yeast as a mitochondrial metallopeptidase (Kaser et al., 2003), found that knockdown of OMA1 in mammalian cells prevents CCCP-induced OPA1 processing (Ehses et al., 2009), while van der Bliek’s group similarly found that OMA1 is an inner-membrane-localized protease that mediates CCCP-inducible L-OPA1 cleavage (Head et al., 2009). OMA1 has since emerged as a major mitochondrial factor for sensing and responding to cellular stress. Subsequently, a range of stimuli have been shown to activate OMA1-mediated OPA1 processing, including oligomycin, ATP depletion (Rainbolt et al., 2016), oxidants (Rainbolt et al., 2015; Garcia et al., 2018), valinomycin, and heat (Baker et al., 2014). OMA1 acts in close cooperation with the *i*-AAA protease YME1L, with YME1L constitutively cleaving L-OPA1 for a steady-state balance of long and short OPA1 isoforms, while OMA1 is stress-activated to complete L-OPA1 cleavage. L-OPA1 is inducibly cleaved by OMA1 at the S1 site, while YME1L cleaves OPA1 at the S2 site (Anand et al., 2014). Cells lacking both YME1L and OMA1 show only L-OPA1 isoforms (Anand et al., 2014), while OMA1 becomes degraded itself following activation by CCCP (Zhang et al., 2014) in a YME1L-dependent manner (Rainbolt et al., 2016). More recently, a YME1L-dependent third OPA1 cleavage site (S3) has been identified (Wang et al., 2020). Mechanistically, OMA1 is localized to the mitochondrial inner membrane (Ehses et al., 2009; Head et al., 2009). The C-terminal M48 domain of OMA1, oriented toward the intermembrane space, is responsible for carrying out OMA1’s proteolytic activity, while the matrix-oriented N-terminal domain appears to play an important role in sensing changes in ΔΨ_m_: OMA1 variants lacking the positively charged N-terminal domain are unable to cleave L-OPA1 in response to loss of ΔΨ_m_ (Baker et al., 2014). Recent work shows that localized fluctuations in ΔΨ_m_, or “flickering,” cause OMA1 activation events as a protective stress response against mitochondrial hyperfusion (Murata et al., 2020), demonstrating a highly sensitive, responsive mode of action. In addition to interacting with YME1L, OMA1 also appears to interact with other inner-membrane factors as part its emerging roles in apoptosis and other cellular stress response pathways.

## OMA1, OPA1, and Cellular Apoptosis

As elegant as the stress-sensitive mechanisms of OMA1-mediated OPA1 proteolysis are, their greater importance to the cell at large is becoming more broadly evident, given their mechanistic involvement in cell-wide stress responses including apoptosis, autophagy, and integrated stress response. A range of cell and organismal studies demonstrate that OPA1 homeostasis directly contributes to apoptosis and other cellular stress pathways, demonstrating a broader impact for mitochondrial dynamics on cellular life and death. Moreover, a growing literature supports developmental roles for OPA1 in cellular differentiation, particularly in energetically demanding contexts such as myocardial and neuronal cell settings.

The arrival of mitochondria as a mechanistic component of apoptosis significantly broadened the organelle’s importance beyond bioenergetics. A variety of apoptotic stimuli activate the release of cytochrome c from the mitochondria to the cytosol, where it activates caspases for apoptotic cell death (Bossy-Wetzel et al., 1998). Strikingly, both loss and proteolytic processing of OPA1 are associated with apoptotic induction. Knockdown of OPA1 causes mitochondrial fragmentation, followed by cytochrome c release and apoptotic induction in HeLa cells (Olichon et al., 2002). Induction of apoptosis via Bim/tBid causes cleavage of L-OPA1 as part of Bax/Bak-mediated apoptosis (Jiang et al., 2014). OPA1’s role in maintaining the cristae formation of the inner membrane allows it to play a role in remodeling the cristae to allow cytochrome c release upon induction of apoptosis (Cipolat et al., 2006), with evidence that this role is functionally distinct from OPA1’s role in inner-membrane fusion (Frezza et al., 2006). OPA1-mediated cristae reorganization is indeed required for Bax-mediated cytochrome c release and apoptosis (Yamaguchi et al., 2008). Consistent with this, a range of findings demonstrate that OMA1 plays a key role in regulating apoptosis (Figure 2). In identifying OMA1 as the stress-responsive protease that cleaves L-OPA1, van der Bliek’s group found that knockdown of OMA1 blunts staurosporine-induced apoptosis (Head et al., 2009). Similarly, pro-apoptotic Bax and Bak, which are recruited to the mitochondria upon apoptotic induction, activate OMA1, while OMA1 knockdown or knockout dramatically attenuates Bim/tBid-induced apoptosis (Jiang et al., 2014).

These cell-based findings are in agreement with organismal findings, in which genetic modification of OMA1/OPA1 homeostasis dramatically impacts physiology. Activation of OMA1 in mouse heart leads to mitochondrial fragmentation and disrupted metabolism, causing dilated cardiomyopathy and heart failure (Wai et al., 2015), while genetic ablation of OMA1 prevents OPA1 cleavage, delaying neuronal apoptosis in a murine neurodegeneration model (Korwitz et al., 2016), as well as mouse models of heart failure (Acin-Perez et al., 2018). Consistent with this, OMA1 silencing confers increased cell proliferation and migration in patient-derived metastatic cancer cells (Daverey et al., 2019), while adenoviral delivery of OPA1 rescues mitochondrial dysfunction in *in vitro* models (Maloney et al., 2020). Taken together, these findings suggest that OMA1’s role in controlling stress-sensitive OPA1 cleavage has crucial importance to cellular fate through modulation of apoptosis.

## New Questions: Molecular Interactions, Apoptotic Priming, and Developmental Roles

The emerging role of OMA1 as a critical stress-sensitive protease responsible for mitochondrial homeostasis, as well as broader cellular stress response, motivates a range of intriguing questions regarding’s interactions and regulation at the inner membrane, as well as the mechanistic contributions of OMA1 to cellular apoptosis. These underlying mechanisms may also have key developmental roles for cells in a variety of lineages, as a small but growing literature indicates that OMA1 and OPA1 are important for differentiation and development.

OMA1’s proteolytic activation and regulatory interactions with other factors represent key areas of mechanistic inquiry for the field. OMA1 interacts with, and is likely to be regulated by, factors including YME1L (Anand et al., 2014; Rainbolt et al., 2016), P32 (Noh et al., 2020), prohibitin (Anderson et al., 2019), and AFG3L2, indicating that the activation of OMA1 within the inner membrane is likely controlled by a complex set of events and interactions within the mitochondrial interior. Within the inner membrane, OMA1 appears to associate as a hexameric oligomer (Levytskyy et al., 2017). While this multimeric OMA1 interacts with the multiple interacting partners described above, it is unclear how this occurs within the inner membrane. Moreover, the N-terminal ΔΨ_m_ sensor domain is oriented on the matrix side of the inner membrane and is required for stress-sensitive proteolytic activity (Baker et al., 2014), but exactly how this positively charged, loosely structured domain senses changes in ΔΨ_m_ and activates OMA1’s proteolytic activity is unclear. To further our working understanding of OMA1 regulation, these fundamental mechanisms, as well as the regulatory effects of the interacting factors, require clarification.

Moreover, the precise molecular mechanisms behind OMA1’s role in “priming” apoptosis remain to be determined: loss of ΔΨ_m_ precedes translocation of Bax to the mitochondria but is not sufficient in and of itself to induce apoptosis (Bossy-Wetzel et al., 1998; Smaili et al., 2001). Furthermore, the precise functional roles of the long and short OPA1 isoforms remain unclear. While L-OPA1 isoforms are clearly required for inner-membrane fusion, recent findings indicate that the cleaved short S-OPA1 isoforms, often thought to be non-functional due to their inability to mediate membrane fusion, actually play roles in maintaining mitochondrial bioenergetics and cristae structure (Lee et al., 2017) and may confer protection against oxidative necrotic cell death (Lee et al., 2020). These findings raise new questions regarding whether the loss of L-OPA1 or the accumulation of S-OPA1 is mechanistically responsible for the apoptotic priming associated with OMA1 activation. This illustrates the range of questions remaining to be resolved regarding the mechanistic impacts of OMA1 and OPA1 on apoptosis in mammalian cells. Similarly, OMA1 is directly involved in activating the cell-wide integrated stress response (ISR). OMA1 cleaves mitochondrially localized DELE1, releasing it to the cytosol, where it interacts with HRI to activate EIF2a, initiating integrated stress response (Fessler et al., 2020; Guo et al., 2020). Loss of OPA1 activates unfolded protein response, associated with age-related muscle loss and inflammation (Tezze et al., 2017), while increased L-OPA1 suppresses mitochondrial autophagy (Lang et al., 2017). Collectively, these findings reveal a central role for OMA1 and OPA1 as mitochondrial stress sensors, with crucial impacts on cell-wide stress response mechanisms.

Given the importance of stress-sensitive OPA1 balance to mitochondrial structure/function balance, as well as cellular apoptosis and stress-response pathways, it is a short leap of logic to envision a role for OPA1 in cellular development. Consistent with this, a modest but growing literature is emerging to reveal key roles for OPA1 in neuronal and cardiac differentiation. Recently, OPA1 was shown to be required for development of GABAergic neurons from embryonic stem cells (Caglayan et al., 2020), while haploinsufficient OPA1 iPSCs demonstrate degeneration of dopaminergic neurons (Jonikas et al., 2018). Gene trapping of OPA1 in murine ESCs causes impaired cardiac differentiation and development (Kasahara et al., 2013). These findings provide intriguing clues to novel OPA1 developmental roles for OPA1 homeostasis, providing a new direction for OPA1’s impact beyond organellar dynamics and apoptosis.

Taken together, these findings reveal that OMA1 and OPA1 control a highly sensitive mechanism for mitochondrial structure/function homeostasis but also play outsized roles in crucial cell-wide signaling pathways including apoptosis and development. A range of intriguing mechanistic questions remain to be answered in characterizing the mechanisms and broader impacts of this mitochondrial “gatekeeper” mechanism. The interactions of OMA1 and OPA1 with an increasing number of inner-membrane proteins and lipids suggest that delineating the higher-order organization of proteases, scaffolding proteins, and interacting lipids within the inner membrane will be critical to effectively understanding the mechanisms of mitochondrial structure/function homeostasis and apoptotic stress response.

## Author Contributions

All authors listed have made a substantial, direct, and intellectual contribution to the work and approved it for publication.

## Conflict of Interest

The authors declare that the research was conducted in the absence of any commercial or financial relationships that could be construed as a potential conflict of interest.
